# A Multicenter Experience from Lebanon in Childhood and Adolescent Acute Myeloid Leukemia: High rate of Early Death in Childhood Acute Promyelocytic Leukemia

**DOI:** 10.4084/MJHID.2015.012

**Published:** 2015-01-01

**Authors:** Roula A. Farah, Jessy G. Horkos, Youssef D. Bustros, Hussein Z. Farhat, Oussama Abla

**Affiliations:** 1 Department of Pediatrics, Division of Hematology/Oncology, Saint George Hospital University Medical Center, Beirut-Lebanon.; 2 Department of Surgery, Saint George Hospital University Medical Center, Beirut-Lebanon.; 3 Department of Laboratory Medicine, University Medical Center Rizk Hospital, Beirut- Lebanon.; 4 Department of Pediatrics, Division of Hematology/Oncology, Toronto Sick Children’s Hospital.

## Abstract

**Background:**

Acute myeloid leukemia (AML) is a disease with marked heterogeneity. Despite major improvement in outcome, it remains a life-threatening malignancy. Demographic and clinical data on pediatric AML is lacking among the Lebanese population.

**Purpose:**

We aimed to identify clinical, molecular and outcome data in children with AML in Lebanon.

**Methods:**

A retrospective chart review of children with AML diagnosed in three Lebanese hospitals during the past 8 years was conducted.

**Results:**

From May 2002 through March 2010, we identified 24 children with AML in Saint George Hospital University Medical Center, University Medical Center Rizk Hospital, and Abou-Jaoude Hospital. Males and females were equally represented; median age at diagnosis was 9 years (range 1–24) and median WBC at diagnosis was 31 × 10^9^/L (range: 2.1–376 × 10^9^/L). Twenty five percent of patients (6 out of 24) had acute promyelocytic leukemia (APL). Karyotype was normal in 33% of patients; t(8;21), inv (16), t(8;9), t(7;11), t(9;11), complex chromosomal abnormality, monosomy 7 and trisomy 8 were the most common cytogenetic abnormalities encountered. Patients were treated on different European and North American protocols. Twelve patients (50%) achieved morphologic CR after cycle 1, 6 of them (50%) had bone marrow relapse within 11 months from diagnosis. Nine patients underwent allogeneic stem cell transplant, and 3 of them are alive at 5 years post-transplant. Early death rate was 16.6% of patients, mainly those with APL and a presenting WBC > 10 × 10^9^/L. Fifty per cent of APL patients had an early death due to DIC despite starting ATRA therapy. Overall, median survival for AML patients who died from disease progression was 25.8 months (range: 1–60 months). Overall disease-free survival was 30.4%. Patients < 10 years of age had a 50% survival rate compared to 0% in patients > 10 years.

**Conclusions:**

Our report highlights the needs in Lebanon for better supportive care of children with APL, including faster ATRA administration and, aggressive transfusions, easy access to stem cell transplant for high-risk AML patients and the need for a national homogenous treatment strategy for children with AML.

## Introduction

Acute myeloid leukemia (AML) is composed of a group of diseases with marked morphological and cytogenetic heterogeneity and accounts for approximately 20% of childhood and adolescent acute leukemias.^1^ The majority of children with AML can achieve complete remission (CR) when treated with conventional chemotherapy. However, despite significant achievements in the treatment of AML, long-term survival remains inferior to childhood acute lymphoblastic leukemia, even if, in high-income countries, intensive therapy in conjunction with adequate supportive care has increased survival rates to ~70%, with event-free survival rates (EFS) of ≥ 50%.^2^

Response to therapy combined with genetic and molecular features are the most important predictors of clinical outcome and are currently used for risk stratification in most clinical trials.^3^ There are no published epidemiologic or clinical data regarding childhood AML in Lebanon. In the present study we aimed to identify clinical, morphological, molecular and outcome data for childhood and adolescent AML in three Lebanese hospitals.

## Methods

We conducted a retrospective chart review of children with AML diagnosed at three Hospitals in Lebanon over the past eight years (Saint George Hospital University Medical Center, University Medical Center Rizk Hospital, and Abou-Jaoude Hospital). Research ethics approval was obtained from each center prior to data collection. Data collected included patient demographics, presenting symptoms and signs, laboratory results at diagnosis, bone marrow morphological, cytogenetic and molecular features as well as treatment details with relapse and survival data.

## Results

**Figure 1:**
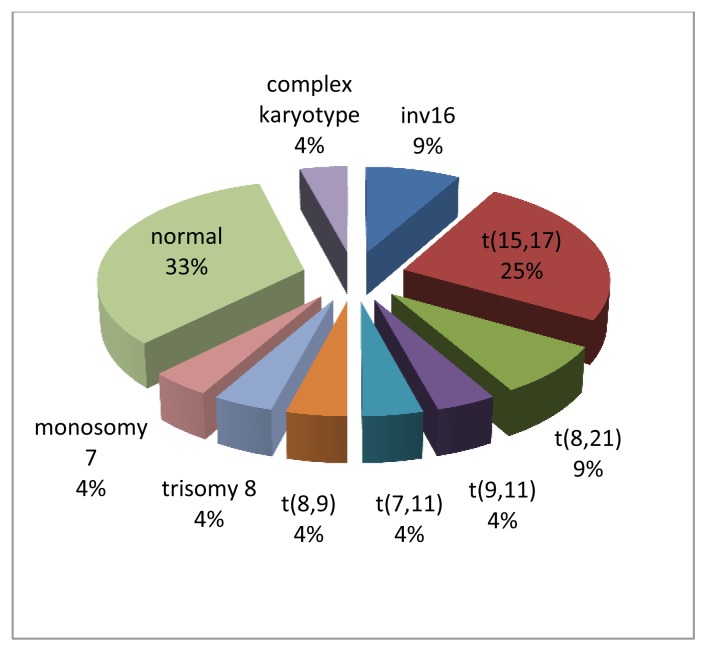
Illustration shows the distribution of chromosomal abnormalities in our AML patients.

Twenty-four patients were diagnosed with AML from May 2002 until March 2010 among the 3 different centers versus thirty nine patients with ALL. Presenting symptoms varied between fever, adenopathy, headache, malaise, bruising, abdominal pain, weight loss, decreased appetite and chloromas. Of the 24 patients, 12 were females (50%) and median age at presentation was 9 years. Seventeen patients (70.8%) were younger than 10 years at diagnosis. The median initial white blood cell count was 31×10^9^/L (range: 2.1–376×10^9^/L). All patients had low hemoglobin and platelet count at diagnosis, with a mean of 9.2 g/dl (range: 4.6–13.3 g/dl) and 46×10^9^/L (range: 10–164×10^9^/L), respectively. Three patients had secondary AML; one was previously treated for Burkitt lymphoma and 2 had Fanconi anemia and were siblings. Regarding the FAB subtypes, M3 was the most frequent (6 of 24; 25%) followed by M4 (5), M1 (4), M0 (2), M2 (2), M6 (2), M7 (1) and 2 patients were not classified. The M0, M1, and M2 subtypes tended to be more common in children > 10 years while the other subtypes were more frequent in those <10 years of age.^4^ M1 was the most frequently observed subtype in five patients with normal karyotype (Table 1). Cytogenetic abnormalities were detected in 16 patients, and normal karyotype was detected in the others. Cytogenetic abnormalities included t(15;17) in 6 children, t(8;21) in 2, inv(16) in 2, t(8;9) in 1, t(7;11) in 1, t(9;11) in 1, complex chromosomal abnormality in 1, monosomy 7 in 1 and trisomy 8 in one child with Down syndrome (Figure 1). *FLT3* gene mutations were detected in 3 patients; one had internal tandem duplication (ITD). However, molecular studies were not performed in all patients, and the real incidence of *FLT3* mutations, as well as other mutations (such as NPM1 or CEBPA), could not be documented.

All patients were treated heterogenously (Table 2) according to either the Berlin-Frankfurt-Munster (BFM)-98 protocol, St Jude AML 97 protocol, the North American CALGB9710/Pediatric Oncology Group (POG)-9421/Children’s Oncology Group (COG)-AAML0531 studies, the French FLA protocol, Associazione Italiana di Ematologia Oncologia Pediatrica (AIEOP)-AML 2002/01 or the Scandinavian NOPHO-AML protocols. Twelve patients (50%) achieved morphologic CR (no minimal residual disease testing was performed), 6 of them (50%) had bone marrow relapse within eleven months from diagnosis. Hematopoietic stem cell transplant (HSCT) was indicated in 15 patients, but only nine underwent the procedure due to economical reasons or lack of a matched donor. Four out of nine had matched related, and 5 had matched unrelated HSCT. Three out of 9 children who underwent HSCT are currently alive at 5 years post-HSCT.

Children with AML < 10 years of age at diagnosis had a 50% survival rate compared to 0% in children > 10 years resulting in worsening prognosis with rising age at presentation.^3^ The p value was 0.05 using Fisher exact test. There was no predilection of gender that affected survival, nor a level of leukocytosis that worsened the prognosis. Among the 15 patients who died, 8 had WBC < 31×10^9^/L, and 7 had WBC > 31×10^9^/L.

Three out of six children (50%) with acute promyelocytic leukemia (APL) had an early death; two died upon initiating of induction due to DIC and CNS bleed, despite a quick diagnosis and early start of all-trans retinoic acid (ATRA), while one died 2 days after diagnosis and before starting any treatment. All 3 patients had an initial WBC > 10^9^/L which is known to be a high-risk feature in APL.

Median survival for patients who died from disease progression was 25.8 months. Overall disease-free survival was 30.4% at 5 years from diagnosis.

## Discussion

Our multicenter study showed a 25% incidence of APL among the pediatric AML cohort, which is higher than that reported in Europe (6–10% rate)^2^ and lower than Latin American countries (37.5% of AML).^5^ The most striking finding in our study was the 50% early death (ED) rate in children with APL. Indeed, 2 out of 6 APL children died due to CNS bleeding despite early initiation of ATRA therapy and one died before starting any treatment. All 3 children had a presenting WBC > 10×10^9^/L. The rate of ED in pediatric APL trials ranged between 3.6% and 7.5%.

In a preliminary analysis of the National Cancer Institute’s Surveillance, Epidemiology and End Results (SEER) database, the ED rate in children (< 18 years) with APL was 8.7%^6^ as opposed to 17.3% to 29% in adult population. In 2 consecutive studies of the PETHEMA Group, the factors significantly prognostic for ED were: abnormal creatinine level, peripheral blood blast cell count exceeding 30×10^9^/L, age older than 60 years, male sex, and WBC exceeding 10×10^9^/L.^7^ However, data on predictors of early death in pediatric APL are lacking.

Another significant finding in our case series was the worse outcome in children with AML aged > 10 years at diagnosis (0% vs 50%; p=0.05), although the numbers are small to draw definite conclusion. In addition, even though the t(8;21) has been usually associated with good prognosis, we could not document this in our study since we had one patient alive post HSCT and one patient died following relapse post-HSCT.

We had one patient with Down syndrome in whom trisomy 8 appeared as a secondary chromosomal change. The prognostic significance of trisomy 8 is controversial.^8^ It has been reported that it does not carry prognostic significance in children except when it appears as a secondary chromosomal change in aggressive cases.^9^ However, our patient lived only 12 months after diagnosis. Furthermore, our study confirmed the worse prognosis in patients with monosomy 7 and treatment-related AML, since both patients from our series died due to progressive disease.

Our study is the first multicenter report on childhood AML in Lebanon, although it does have a few limitations. The analysis is mainly retrospective with a small number of patients who were treated on heterogeneous chemotherapy protocols. Some high-risk patients fared poorly likely due to the inability to undergo HSCT and might have had a better outcome with HSCT. In addition, our data is limited to three centers and does not reflect all the Lebanese pediatric AML population. However, the study is important since it indicates the need for faster access to transplant for high risk AML patients and national homogenous treatment strategies in Lebanon. Further, the very high early death rate observed in our APL patients should stimulate an immediate change of practice in our country for this subgroup since most of the early deaths were related to DIC. Rapid administrations of ATRA and aggressive platelet and fibrinogen replacement have been found to reduce early death in APL, and these supportive measures should be rapidly implemented. In addition, small pediatric studies from China and India have confirmed the therapeutic efficacy of front-line single-agent arsenic trioxide (ATO) therapy, which was comparable to historical ATRA plus chemotherapy regimens.^10^,^11^ Future directions in pediatric APL trials will focus on prospectively testing the front-line combination of ATRA and ATO, which is the new standard of care for standard-risk APL in adults.^12^

## Figures and Tables

**Table 1:** Classification of AML patients within age groups by sex, FAB classification, and chromosomal abnormalities

Category	Total	Age ≤ 10 years	Age > 10 years
**Sex**			
**Male**	12	10	2
**Female**	12	7	5
**Total**	24	17	7
**FAB Classification**			
**M0**	2	1	1
**M1**	4	2	2
**M2**	2	1	1
**M3**	6	5	1
**M4**	5	3	2
**M5**	0	0	0
**M6**	2	2	0
**M7**	1	1	0
**Balanced Abnormalities**			
**t(9;11)**	1	1	0
**t(8;21)**	2	1	1
**t(15;17)**	6	5	1
**Inv (16)**	2	1	1

**Table 2:** Outcome results

FAB subtype	Number of patients	Treatment	Outcome
M0	2	(AIEOP)-AML 2002/01	One dead and one alive
M1	4	(COG)-AAML0531 (BFM)-98 protocol	Three dead and one alive
M2	2	(BFM)-98 protocol (COG)-AAML0531	One dead and one alive
M3	6	(BFM)-98 protocol CALGB9710	Three dead and three alive
M4	5	FLA protocol COG)-AAML0531 (POG)-9421 (BFM)-98 protocol	Three dead, one alive and one lost to follow up
M5	0		
M6	2	NOPHO-AML protocol	One dead and one alive
M7	1	St Jude AML 97 protocol	Dead
Non classified	2	(COG)-AAML0531	Dead
